# Molecular Characterization and Chemotactic Function of CXCL8 in Northeast Chinese Lamprey (*Lethenteron morii*)

**DOI:** 10.3389/fimmu.2020.01738

**Published:** 2020-08-18

**Authors:** Xinyun Zhu, Zhe Zhang, Jianfeng Ren, Liang Jia, Shaoqing Ding, Jiafei Pu, Wenyuan Ma, Yan Tao, Yao Zu, Weiming Li, Qinghua Zhang

**Affiliations:** ^1^Key Laboratory of Exploration and Utilization of Aquatic Genetic Resources, Ministry of Education, Shanghai Ocean University, Shanghai, China; ^2^International Research Center for Marine Biosciences, Ministry of Science and Technology, Shanghai Ocean University, Shanghai, China; ^3^Key Laboratory of Freshwater Aquatic Genetic Resources, Ministry of Agriculture, Shanghai Ocean University, Shanghai, China; ^4^College of Food Science and Technology, Shanghai Ocean University, Shanghai, China; ^5^Department of Fisheries and Wildlife, Michigan State University, East Lansing, MI, United States

**Keywords:** *Lethenteron morii*, neutrophils, chemokine, CXCL8, chemotaxis

## Abstract

Chemokine-induced chemotaxis of leukocytes is an important part of the innate immunity and has been shown to mediate inflammation in all groups of jawed vertebrates. For jawless vertebrates, hagfish leukocytes are known to show chemotaxis toward mammalian complement anaphylotoxin and Gram-negative bacteria lipopolysaccharide. However, whether chemokines mediate chemotaxis of leukocytes in jawless vertebrates has not been conclusively examined. Here, we show C-X-C motif chemokine ligand 8 (CXCL8, also named interleukin 8) of the Northeast Chinese lamprey (*Lethenteron morii*) (designated as LmCXCL8) induces chemotaxis in its leukocytes. We identified LmCXCL8 and found it possesses the characteristic N-terminal cysteine residues and GGR (Gly-Gly-Arg) motif. The *Lmcxcl8* gene was found to be expressed in all examined tissues, and its expression was inducible in the lamprey challenged by an infectious bacterium, *Pseudomonas aeruginosa*. A recombinant LmCXCL8 protein elicited concentration-dependent chemotaxis in peripheral blood leukocytes isolated from the Northeast Chinese lamprey. Based on these results, we conclude that LmCXCL8 is a constitutive and inducible acute-phase cytokine that mediates immune defense and trace the chemotactic function of chemokine to basal vertebrates.

## Introduction

Chemokine is a group of cytokines that induce chemotaxis of white blood cells, which is critical to the inflammatory process and innate defense against pathogens. Chemokines have been reported in species across the whole vertebrate clad, from agnathans to mammals (1, 2). Where studied, chemokines have been found to play a critical role in regulating immune cell migration under both inflammatory and normal physiological conditions (3). According to their function, chemokines can be divided into inducible or inflammatory chemokines and constitutive or homeostatic chemokines (4). Chemokines promote leukocyte mobilization and regulate inflammatory responses and also regulate differentiation of recruited cells, as well as development of the immune system (5–9). Based on the arrangement of the first two highly conserved N-terminal cysteine residues, chemokines are classified into subfamilies, CXC, CC, C, CX3C, and CX (5). Genes encoding these chemokines have been found in the genomes of all vertebrate groups. Up to date, river lamprey CXCL8 (10), Japanese lamprey CXCL17 (11), and sea lamprey CXCL17 (12) have been documented. However, in contrast to extensive studies on functions of chemokines in jawed vertebrate (13), examination of chemokine functions was limited in basal vertebrates and invertebrates.

Among CXC chemokines, C-X-C motif chemokine ligand 8 (CXCL8), also named interleukin 8 (IL-8), was first identified in human in 1987 (5) and shown to activate massive chemotactic responses in inflammation. It is secreted by a wide range of cells including macrophages, monocytes, and epithelial and endothelial cells (14, 15). Numbers of CXCL8 transcripts increase dramatically after stimulation by bacteria (16–18), virus (19, 20), and certain cytokines such as IL-1 and tumor necrosis factor (TNF) (21). Structures of CXCL8 have been determined species across vertebrate groups, including human (*Homo sapiens*) (22), chicken (*Gallus gallus*) (23), frog (*Xenopus laevis*) (24), zebrafish (*Danio rerio*) (25), and river lamprey (*Lampetra fluviatilis*) (10). However, no reported study has indicated that mice express CXCL8. The chemotactic activity of CXCL8 has also been demonstrated in a few fish species, such as rainbow trout (*Oncorhynchus mykiss*) (26), half-smooth tongue sole (*Cynoglossus semilaevis*) (27), common carp (*Cyprinus carpio*) (28), flounder (*Paralichthys olivaceous*) (29), and large yellow croaker (*Larimichthys crocea*) (30). As reported, there is one lineage of CXCL8 with an N-terminal tripeptide motif glutamate-leucine-arginine (ELR) preceding the CXC motif, and the ELR motif is essential for attracting neutrophils but not for lymphocytes and monocytes in mammalian species. However, there are three distinct CXCL8 lineages in fish species, including CXCL8-L1, CXCL8-L2, and CXCL8-L3 (31). Further, fish species CXCL8 usually has an XXR motif, not an ELR motif, and the incomplete ELR motif does not affect fish CXCL8 neutrophil recruitment function (32). In previous reports, the ELR in mammals or XXR in fish are evolved from GGR of lamprey (31). Whether the origin of leukocyte chemotaxis response induced by CXCL8 is from lamprey needs to be further examined.

The lamprey and hagfish are two groups of vertebrates that share a unique phylogenetic position at the interface between invertebrates and the vertebrate with hinged jaws and are considered as useful models for studying the evolution of immunity (33). The identification of CXCL8 and the existence of leukocytes in lamprey provide a model to infer the evolutionary origin of leukocyte chemotaxis response induced by CXC chemokine. In this study, we aim to use lamprey as a model to trace the chemotaxis responses of leukocytes to CXC chemokine back to jawless vertebrate. Here we report the molecular characteristics and chemotactic function of CXCL8 in Northeast Chinese lamprey (LmCXCL8).

## Materials and Methods

### Animals Care and Maintenance

Feral Northeast Chinese lampreys were sampled in tributaries of the Yalu River, Dandong City, Liaoning province, China. They were raised at 8°C in the freshwater. Embryos were produced through artificial fertilization and incubated at 18°C and hatched on the 10th day. Samples were collected between 5 and 20 days after fertilization (dpf). Lampreys were handled according to the procedures of the Institutional Animal Care and Use Committee of Shanghai Ocean University, Shanghai, China. The proposed research methodology received clearance from the Shanghai Ocean University Experimentation Ethics Review Committee (SHOU-DW-2016-003).

### Cloning and Sequence Analysis of Full-Length *Lmcxcl8* cDNA

Adult Northeast Chinese lampreys were euthanized in 0.2% MS 222 (A5040; Sigma-Aldrich, USA), and their intestine removed and separately treated with Trizol (15596026; Invitrogen, USA). RNA was isolated according to the manufacturer's instruction. The cDNA was synthesized from 1 μg of RNA with PrimeScript RT Master Mix (RR036A; TaKaRa, Japan) and subjected to polymerase chain reaction (PCR) under the following conditions: an initial denaturation step of 2 min at 94°C, followed by 35 cycles of denaturation at 98°C for 10 s, annealing at 60°C for 30 s and 30 s of extension at 72°C, and final elongation step at 72°C for 5 min, using primers CXCL8-F1 and CXCL8-R1 (Table 1). The primer pair was designed based on the partial cDNA sequence of Japanese lamprey genome (http://jlampreygenome.imcb.a-star.edu.sg/), which was largely homologous to CXCL8 chemokines in mammals and other fish species. The amplicon cDNA fragments were ligated into pMD19-T vector, transformed into *Escherichia coli* competent cells DH5α and subsequently sequenced (Shanghai Sangon Biotech, China). The cDNA sequence of *cxcl8* was analyzed using BLAST at the NCBI server (http://www.ncbi.nlm.nih.gov/BLAST). Signal peptide prediction was made using SignalP 4.1 Server (http://www.cbs.dtu.dk/services/SignalP/).

**Table 1:** Primers used in the study.

**Name**	**Sequence (5^**′**^-3^**′**^)**	**Used for**	**PCR product (bp)**
CXCL8-F1	GTG GAG GGG AGC GAC AAG ACG	Cloning	1,055
CXCL8-R1	TTT TTG TTT TAG AAT TTT TT	Cloning	
CXCL8-F2	ATC CAT CCC AAG CAT TTC CAG	qPCR	144
CXCL8-R2	GCT CAT CAC CTT CCT CAC CCA G	qPCR	
β-Actin-F1	CCA CCA TGA AGA TCA AGA T	qPCR	112
β-Actin-R1	CTG TTG CTG ATC CAC ATC	qPCR	
pCold-CXCL8-F	GGA ATT CCA TA TGT CTA TCT TCG AAGGTG	Cloning	237
pCold-CXCL8-R	CCC AAG CTT TTA CGG GGT CGG TTT CGG G	Cloning	
CXCL17-F1	GGC AAA GGA AAA GAC ATC A	qPCR	118
CXCL17-R1	GAC ACC CAT CAC ACA GAC AG	qPCR	

CXCL8 amino acid sequences were downloaded from NCBI and used to construct a phylogenetic tree. The sequences of coelacanth and spotted gar were identified using BLASTP from their genome assemblies in Ensembl genome browser 91 (http://asia.ensembl.org/index.html) and confirmed using gene tree function in Ensemble. The phylogenetic tree was constructed using program MEGA 6 (34) with neighbor-joining algorithm with 2,000 bootstrap replications. Human CXCL6 (P80162), mouse CXCL6 (GenBank: ABG81953.1), human CXCL15 (GenBank: NP_000576.1), mouse CXCL15 (GenBank: EDL10891.1), zebrafish CXCL15 (GenBank: NP_001034654.1), human CXCL17 (GenBank: Q6UXB2), mouse CXCL17 (GenBank: Q5UW37), and Japanese lamprey CXCL17 (GenBank: BAF93839.1) were used as outgroups.

### Tissue Distribution of *Lmcxcl8* Transcripts in Juvenile and Adult Lampreys

Various tissues, including the brain, intestine, heart, gills, muscle, liver, kidney, supraneural body (SB), skin, and nose, were collected from six healthy juveniles and six healthy adults. The juvenile was 10 ± 2 cm total length, and the adult was 30 ± 5 cm total length. RNA was isolated and reverse transcribed into first-strand cDNA from 1 μg of RNA. Real-time PCR was performed with a gene-specific primer set of CXCL8-F2 and CXCL8-R2. β*-Actin* was amplified as an internal control with the primer set of β-actin-F1 and β-actin-R1 (Table 1). Real-time PCR was performed on LightCycler 480 II using LightCycler 480 SYBR Green I Master (4707516001; Roche, Switzerland). Cycling conditions were 95°C for 10 min, followed by 40 cycles of 95°C for 10 s and 60°C for 30 s. Each reaction was carried out in triplicates, and a melting curve analysis was performed to confirm the specificity of the reactions. The average ΔCT value was calculated by subtracting the average β*-actin* CT from the average target gene CT. The −Δ*ΔCT* was calculated by subtracting the control ΔCT from the treatment ΔCT. The relative quantity of mRNA was calculated as 2^−(ΔΔ*CT*)^. Relative quantity minimum and maximum were calculated for a 95% confidence interval using the Student *t*-value and the variance of the target CT average.

### Induction of *Lmcxcl8* and *Lmcxcl17* Expression in Immune Tissues in Larva and Adult Lampreys

Bacterial challenge was carried out with *P. aeruginosa* strain PA11, which was isolated from diseased adult Northeast Chinese lamprey, stored at −80°C. It was incubated to midlogarithmic stage at 28°C in nutrient broth (NB) medium and serially diluted with sterile H_2_O by 10^6^-fold. The dilutions for the culture were plated on Nutrient Agar (NA) medium and incubated for 24 h at 28°C before counting. The number of colony-forming unit (CFU) was then recorded. The bacteria cells for final experiment were harvested by centrifugation at 5,000 revolutions/min, washed thrice with sterile normal saline (NS), and finally resuspended in NS at the appropriate concentration. The larval lampreys at 20 dpf were collected and raised at 18°C. A group of 10 larval lampreys was soaked in the sterile water consisting of 1.65 × 10^6^ CFU/mL of *P. aeruginosa*. Another group of 10 larval lampreys was kept in the sterile water as a control. Larvae were monitored every 12 h, with dead or moribund larvae removed throughout the incubation. Total RNA was extracted from six larvae sampled at 0, 24, 48, 72, and 96 h of exposure, as well as the control. The cDNA was synthesized from 1 μg of RNA for real-time PCR. The *Lmcxcl17* gene-specific primer pair (CXCL17-F1 and CXCL17-R1; Table 1) was designed based on the *cxcl17* cDNA sequence of Japanese lamprey (GenBank: AB303391.1).

The adult lampreys were maintained with a flow-through freshwater supply at 8°C. After being acclimated for 7 days, the healthy lampreys were used for the challenge experiments. A group of 24 adult lampreys was each injected intraperitoneally with bacterial suspension consisting of 1.96 × 10^6^ CFU/mL of *P. aeruginosa* at a dose of 500 μL/100 g body mass. Another group of 24 lampreys was injected with sterilized NB medium as a control. The RNAs of intestine, kidney, SB, and gill were extracted from adults sampled at 0, 6, 12, 24, 48, and 72 h postinfection. The cDNA was synthesized from 1 μg of RNA for real-time PCR.

### Expression and Purification of Recombinant LmCXCL8 Protein

The synthetic gene encoding the LmCXCL8, codon-optimized for *E. coli* expression, was ordered from TaKaRa. The LmCXCL8 was expressed as a 6× His-tagged fusion protein using a pCold I vector (3360; TaKaRa, Japan). Briefly, the LmCXCL8 gene fragment without signal peptide sequence was amplified with the primer set of pCold-CXCL8-F and pCold-CXCL8-R (Table 1) and cloned into the *Nde*I/*Hin*dIII–digested pCold I vector. The constructed plasmid was transformed into *E. coli* BL21 competent cells (9120; TaKaRa, Japan), and the pCold I vector was used as a control. The colonies containing transformants were confirmed by DNA sequencing. The recombinant LmCXCL8 protein expression was induced by 0.4 mM IPTG (TaKaRa, Japan, 9030), and bacteria were incubated in 400 mL LB broth base (12780052; Invitrogen, USA) for 22 h at 15°C while shaking. The resulting bacterial pellets were resuspended in phosphate-buffered saline (PBS) buffer and sonicated 60 times for 5 s with pauses on ice for 5 s until clear lysates were obtained. After centrifugation at 12,000 g for 10 min, the recombinant LmCXCL8 protein in supernatant was purified using Bio-Scale™ Mini Profinity™ IMAC Cartridge through Profinia™ Protein Purification Instrument (732-4610; Bio-Rad, USA). The purified recombinant LmCXCL8 protein was analyzed using sodium dodecyl sulfate–polyacrylamide gel electrophoresis (PAGE) with Coomassie brilliant blue and Western blotting with anti-6 × His tag® antibody (ab18184; Abcam, UK). We identified the protein with matrix-assisted laser desorption/ionization–time of flight (TOF)/TOF method (Shanghai Applied Protein Technology, China).

### Chemotaxis Assay

Peripheral blood leukocytes (PBLs) from adult lampreys were prepared from the blood sampled through cardiac puncture and separated using density gradient centrifugation with Percoll solution (WBC1080F; TBD Science, China) as reported (35). The leukocytes were adjusted to 1 × 10^6^ cells/mL in L-15 medium (11415056; Gibco, USA) for the migration assay. The chemotaxis assay was performed with a 24-well Costar Transwell apparatus with 3.0-μm-pore polycarbonate membrane insert (3415; Corning, USA). A 100-μL aliquot of leukocytes (1 × 10^6^ cell/mL) was added to the Transwell upper chamber. Purified recombinant LmCXCL8 protein was diluted in L-15 medium to 10, 50, 100, 200, 300, and 500 μg/mL. At the same time, we used rabbit LmCXCL8 polyclonal antibody (538-1; Youke, China) to block 100 μg/mL of recombinant CXCL8 protein at room temperature for 1 h. Then a 600-μL aliquot of each dilution of protein was added to the Transwell lower chamber, with PBS as a control. The plates were incubated at 18°C for 4 h. The number of cells that migrated into the lower chamber was counted under an Axio Observer Z1 microscope (Zeiss, Germany). The assay procedure was repeated independently three times. Chemotactic activity was defined as a chemotactic index or the number of cells that migrated in response to recombinant LmCXCL8 protein or PBS divided by the number of cells that migrated to the L-15 medium (negative control). The cells that migrated into the lower chamber were photo recorded.

### Statistical Analysis

All data were analyzed with GraphPad Prism 5.0 software. The significance test for induction of *Lmcxcl8* and *Lmcxcl17* expression in immune tissues in larva and adult lampreys was determined by the one-way analysis of variance (ANOVA), and Tukey multiple-comparisons test for residuals followed normal distribution and equal variance. Residuals did not follow normal distribution Kruskal–Wallis test and Dunn multiple-comparisons test. The significance test of the chemotactic activity between the experimental and control (PBS) groups was determined using Brown–Forsythe and Welch ANOVA test and Dunnett T3 multiple-comparisons test (residuals that follow normal distribution but not equal variance).

## Results

### Molecular Characteristics of LmCXCL8

**Figure 1:**
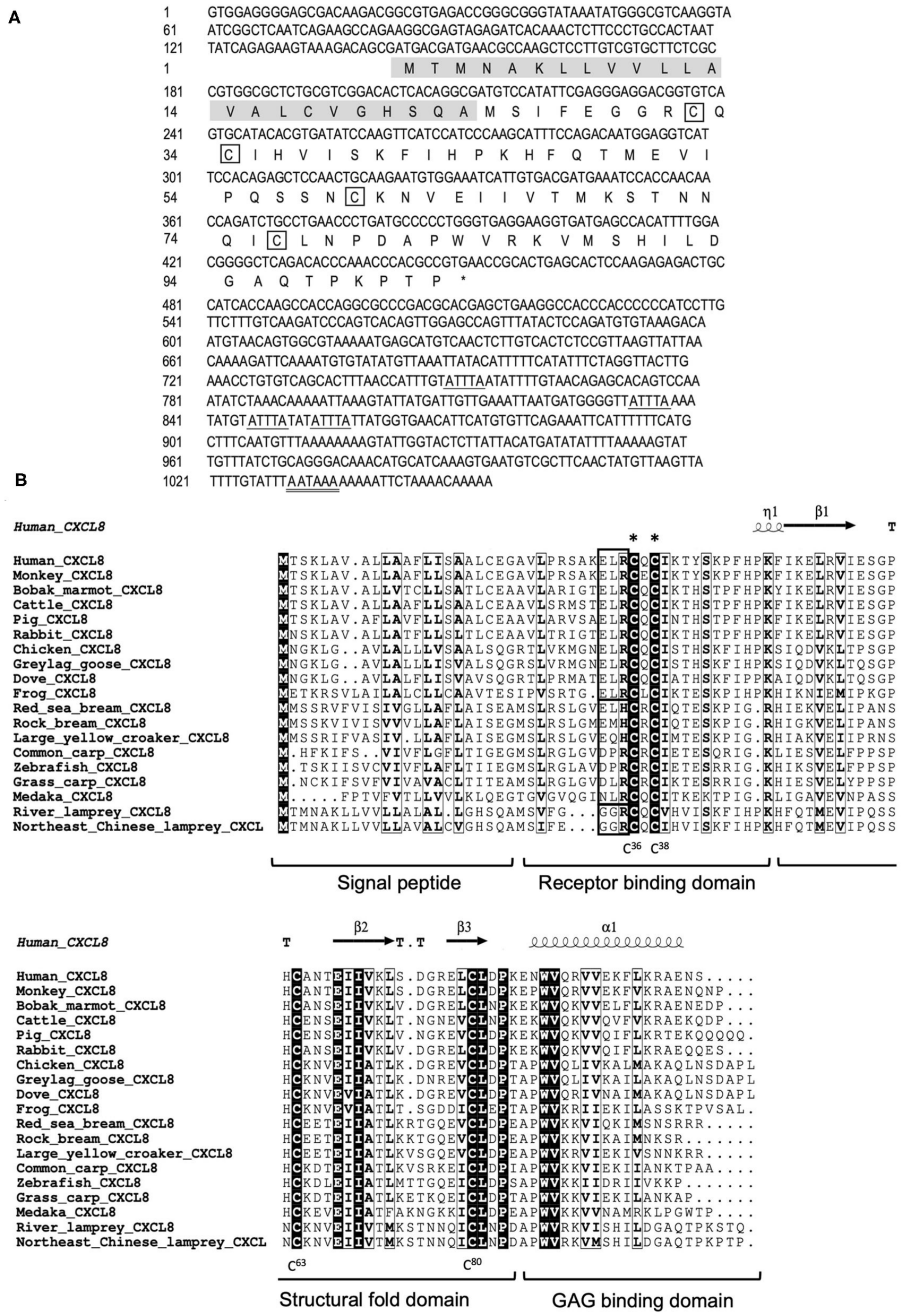
Structural characteristics of LmCXCL8. **(A)** Nucleotide and deduced amino acid sequences of LmCXCL8. In the predicted sequence, the signal peptide is shaded (residues 1–23), whereas the four conserved cysteine residues are boxed. The stop codon (TGA) is represented with an asterisk. The motif associated with mRNA instability (ATTTA) is indicated with a single underline, and the typical polyadenylation signal (AATAAA) is indicated with a double underscore. **(B)** Alignment of the LmCXCL8 predicted sequences with CXCL8 of other species. The four conserved cysteine residues in it (positions in lamprey are highlighted below). The CXC motifs are marked with asterisks. The ELR motifs are boxed (bold black). The highly conserved residues are shown in light black boxes (90% sequence identities). The secondary structures were marked on the top of the sequences according to the x-ray structure of human CXCL8 (PDB: 4XDX). Turns mark as T. 3_10_-helix mark as η. α-helix mark as α. β sheets mark as β (36). CXCL8 function domains are marked under the alignment.

We cloned the full-length cDNA of *Lmcxcl8* gene from Northeast Chinese lamprey (accession no. KY379068), which was 1,055 nt in length, including a 5′-untranslated region (UTR) of 142 nt and a 3′-UTR of 604 nt. There were four mRNA instability motifs (ATTTA) and a polyadenylation signal (AATAAA) in the 3′-UTR. The 309-nt open reading frame encodes a protein of 102-amino-acid residues (aa) with the predicted molecular weight of 11.23 kDa and isoelectric point of 9.37. The predicted protein consists of a 23-aa signal peptide and a 79-aa mature polypeptide (Figure 1A). The primary LmCXCL8 sequence was compared with those of river lamprey, human, monkey (*Macaca mulatta*), bobak marmot (*Marmota monax*), cattle (*Ictalurus punctatus*), pig (*Sus scrofa*), rabbit (*Oryctolagus cuniculus*), chicken (*Gallus gallus*), graylag goose (*Anser anser*), dove (*Columba livia*), red sea bream (*Pagrus major*), rock bream (*Oplegnathus fasciatus*), grass carp (*Ctenopharyngodon idella*), common carp, zebrafish, medaka (*Oryzias latipes*), large yellow croaker, and frog. The LmCXCL8 protein has the typical arrangement of four conserved cysteine residues as found in other chemokines (Figure 1B, positions C^36^, C^38^, C^63^, and C^80^). Similar to fish CXCL8, LmCXCL8 lacks the complete ELR motif of mammals and birds and has a GGR motif the same as that of river lamprey CXCL8 (10) (Table 2).

**Table 2:** ELR motif analysis of CXCL8.

	**Species**	**Common name**	**Motif**	**Author**	**Year**	**Accession no**.
Mammals	*Homo sapiens*	Human	ELR	Schmid	1987	P10145.1
	*Oryctolagus cuniculus*	Rabbit	ELR	Yoshimura	1991	P19874.2
	*Sus scrofa*	Pig	ELR	Lin	1994	P26894.1
	*Macaca mulatta*	Monkey	ELR	Villinger	1995	P67813.1
	*Bos taurus*	Cattle	ELR	Morsey	1996	P79255.1
	*Marmota monax*	Bobak marmot	ELR	Huang	2007	ABY67262.1
Birds	*Gallus gallus*	Chicken	ELR	Sugano	1987	P08317.1
	*Anser anser*	Graylag goose	ELR	Wu	2008	ABD49205.1
	*Columba livia*	Dove	ELR	Wu	2008	ABD49206.1
Amphibian	*Xenopus laevis*	Frog	ELR	Cui	2013	AEB96252.1
Fish	*Melanogrammus aeglefinus*	Haddock	ELR	Corripio-Miyar	2007	CAD97422.2
	*Gadus morhua*	Atlantic cod	ELR	Seppola	2008	xABV59376.1
	*Oncorhynchus mykiss*	Rainbow trout	DLR	Laing	2002	CAC83945.1
	*Ctenopharyngodon idella*	Grass carp	DLR	Wang	2013	AEM05971.1
	*Danio rerio*	Zebrafish	DPR	DeVries	2005	CCQ71734.1
	*Cyprinus carpio*	Common carp	DPR	Abdelkhalek	2009	BAH98111.1
	*Takifugu rubripes*	Fugu	EQH	Saha	2007	BAD26621.1
	*Larimichthys crocea*	Large yellow croaker	EQH	Wan	2009	AKM12660.1
	*Paralichthys olivaceous*	Japanese flounder	SLH	Lee	2001	AAL05442.1
	*Ictalurus punctatus*	Catfish	AER	Chen	2005	AKQ06246.1
	*Pagrus major*	Red sea bream	ELH	Jin	2013	AHC69388.1
	*Oplegnathus fasciatus*	Rock bream	EMH	Jin	2013	AHC69385.1
	*Oryzias latipes*	Medaka	NLR	Unknown	2015	XP_004065776.1
Cyclostomata	*Lampetra fluviatilis*	River lamprey	GGR	Najakshin	1999	CAA13114.1
	*Lethenteron morii*	Northeast Chinese lamprey	GGR	This study	2017	KY379068

### Phylogeny of Lamprey CXCL8

**Figure 2:**
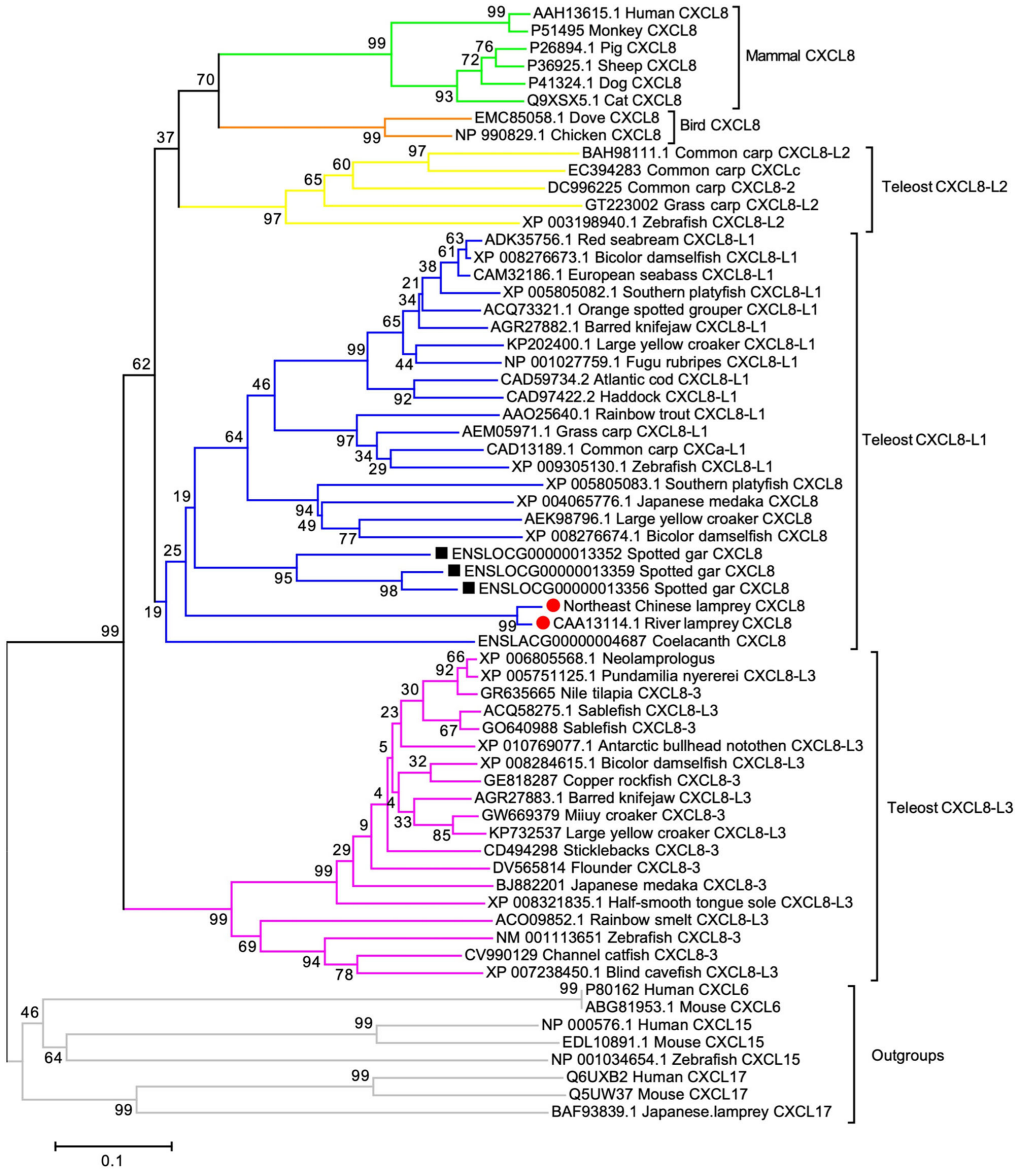
Phylogenetic analysis of CXCL8. The phylogenetic tree was constructed using MEGA 6 with the full length of CXCL8 amino acid sequences, which were analyzed using the neighbor-joining algorithm (bootstrap, 2,000 iterations). Clades of teleost CXCL8-L1, CXCL8-L2, and CXCL8-L3 and clades of lamprey, Gar, bird mammal CXCL8 are marked aside. Each branch name is composed of sequence ID and species common name. Lamprey CXCL8 are highlighted solid red circles. Gar CXCL8 is highlighted by solid black squares. Node support rates are marked. Scale bar, the percentage of genetic variation (0.1 is 1%).

To determine if the cloned LmCXCL8 is homologous to CXCL8 of other vertebrate species, we constructed a phylogenetic tree by using CXCL6, CXCL15, and CXCL17 as outgroups. With shared amino acid sequence identity at 90.2%, LmCXCL8 and river lamprey CXCL8 (10) formed a lamprey CXCL8 clade that separated clearly from outgroups (Figure 2). These results confirmed the homologous of LmCXCL8 to vertebrate CXCL8.

In order to infer the evolutionary position of LmCXCL8 in the phylogenetic tree, we added CXCL8 from lampreys, lobe-finned fish, ray-finned fish, teleosts (1, 30), birds, and mammals. The lobe-finned fish coelacanth (*Latimeria chalumnae*) and ray-finned fish spotted gar (*Lepisosteus oculatus*) were included in the phylogenetic analyses because these two species diverged after the two rounds of early vertebrate genome duplication (VGD1 and VGD2) and before the teleost genome duplication, which may help infer the evolutionary position of LmCXCL8 further. One CXCL8 was determined in coelacanth, and three CXCL8 that ranged in tandem repeat patterns were found in spotted gar. In our phylogenetic tree, consistent with a previous report (30), teleost CXCL8 formed three groups, CXCL8-L1, CXCL8-L2, and CXCL8-L3. Spotted gar CXCL8 were clustered together as an outbranch of teleost CXCL8-L1, lamprey CXCL8 as an outbranch of spotted gar CXCL8, and the coelacanth CXCL8 as an outbranch of lamprey CXCL8. Together, spotted gar, lamprey, and coelacanth CXCL8 clustered with teleost CXCL8-L1 and separated from teleost CXCL8-L2 and mammalian CXCL8, which is consistent with the phylogenetic tree in a previous report that suggests the ELR in mammalian or XXR in fish are evolved from GGR of lamprey (31).

### Constitutive and Inducible Expression of *Lmcxcl8*

**Figure 3:**
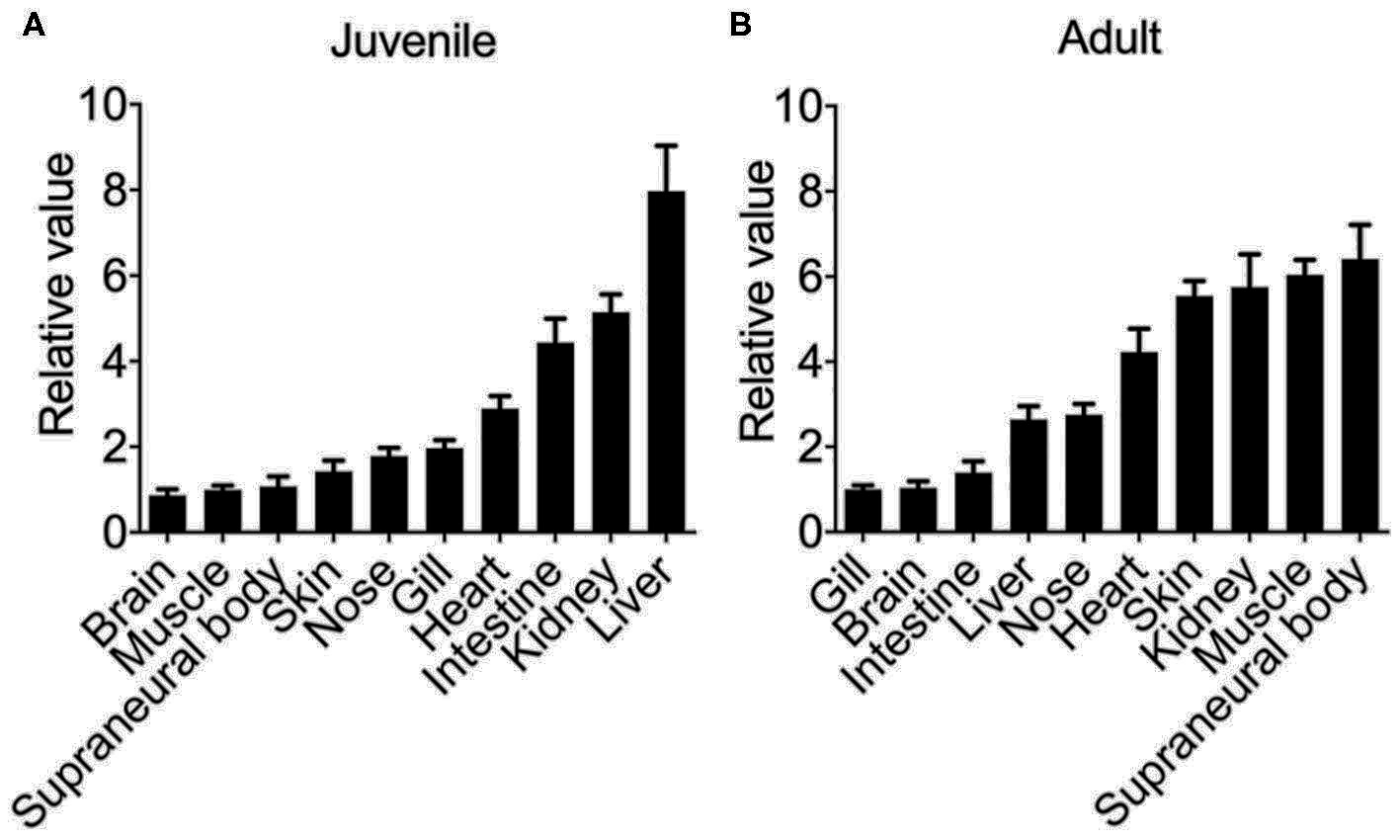
*Lmcxcl8* transcript levels in various tissues in juvenile **(A)** and adults **(B)**. β*-Actin* transcription was used as an internal control for real-time PCR. Bars show mean ± SEM of results from six lampreys pooled from three independent experiments.

First, we examined the constitutive tissue distribution of *Lmcxcl8* in juvenile and adult lamprey using real-time PCR. Analysis of *Lmcxcl8* transcripts in different tissues revealed distinct expression patterns (Figure 3). In the juveniles, *Lmcxcl8* transcripts were detected in 10 tissues including liver, kidney, intestine, heart, gills, nose, skin, SB, muscle, and brain, with the highest *Lmcxcl8* level found in the liver and the lowest in the brain (Figure 3A), whereas in the adults *Lmcxcl8* transcripts were detected in, in descending order, SB, muscle, kidney, skin, heart, nose, liver, intestine, brain, and gills (Figure 3B). *Lmcxcl8* was found in all tissues examined in juvenile and adult lampreys.

**Figure 4:**
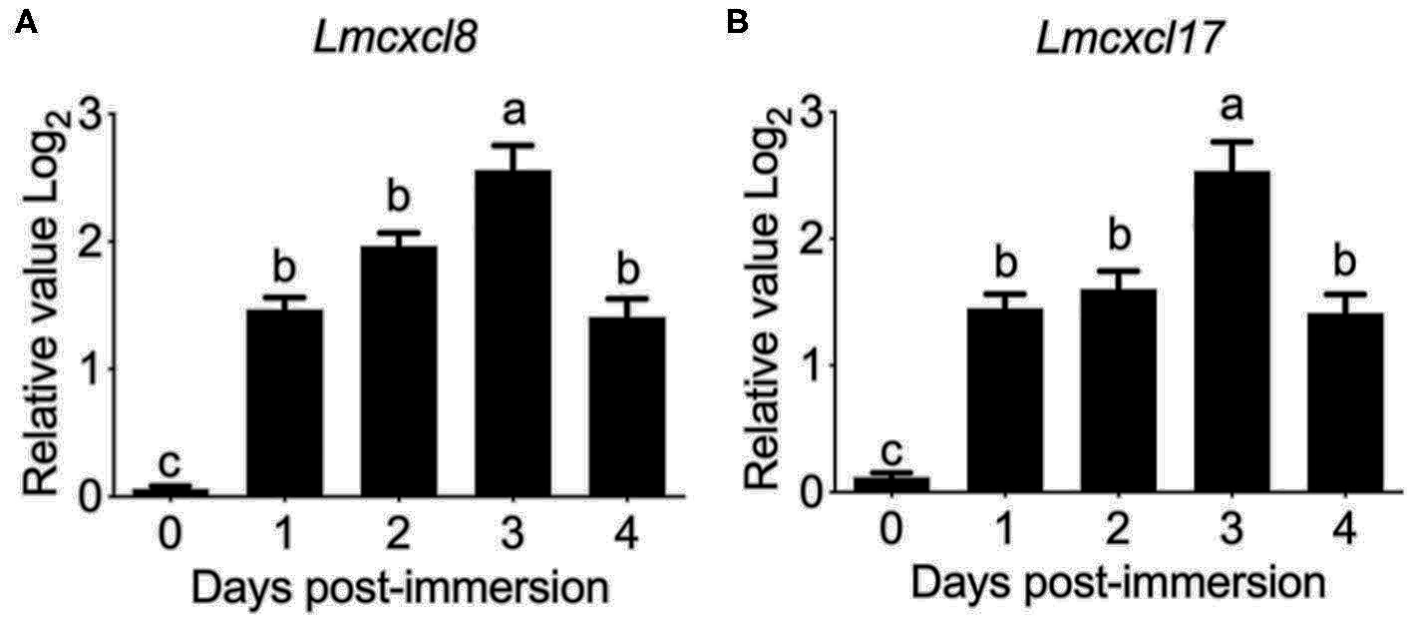
Time courses of *Lmcxcl8* and *Lmcxcl17* expression in 20 dpf larval Northeast Chinese lamprey changed with *P. aeruginosa*. The larvae were maintained in water with 1.65 × 10^6^ CFU/mL of *P. aeruginosa*. Transcript numbers were normalized against those of unchallenged samples. **(A)** Expression of *Lmcxcl8* detected from larvae after the bacterial challenge. **(B)** Expression of *Lmcxcl17* detected from larvae after the bacterial challenge. β*-Actin* transcription was used as an internal control for real-time PCR. Bars show mean + SEM of results from 10 lampreys pooled from three independent experiments. One-way ANOVA, *p* < 0.0001; lowercase letters above each bar denote statistical results from Tukey multiple comparisons: the bars sharing the same letter represent means that are not different from each other (*p* > 0.05), whereas the bars labeled with different letters indicate means that are different (*p* < 0.05).

Previous studies have shown that tissue expression patterns of CXCL8 are largely influenced by species or pathological status (30). Because inducible expression is a hallmark feature of CXCL8, we also examined the modulation of *Lmcxcl8* expression in Northeast Chinese lamprey challenged by *P. aeruginosa*. In larval lampreys (20 dpf) immersed in the bacterial suspension for a duration of 4 days, the expression of *Lmcxcl8* was notably upregulated from the first day after the challenge was commenced. The mRNA levels peaked on the third day and started to decrease on the fourth day (Figure 4A).

To further confirm the expression pattern of *Lmcxcl8*, we also examined transcripts of *Lmcxcl17* in the infected lamprey. *Lmcxcl17* encodes a proinflammatory mucosal chemokine and has been shown to be inducible by lipopolysaccharide in the Japanese lamprey (*Lethenteron japonicum*) (11). In this study, the expression pattern of *Lmcxcl17* was virtually identical to that of *Lmcxcl8* (Figure 4B).

**Figure 5:**
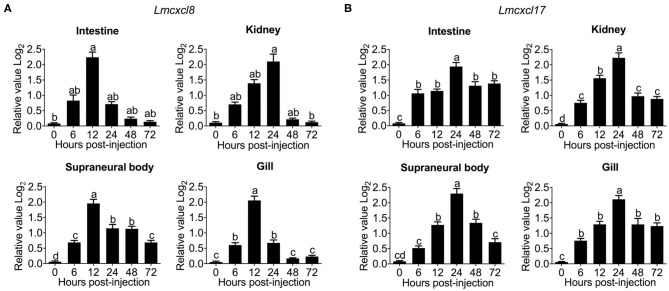
Time courses of *Lmcxcl8* and *Lmcxcl17* expression in adult Northeast Chinese lamprey challenged with *P. aeruginosa*. The adult lampreys were injected intraperitoneally with of *P. aeruginosa* solution (1.96 × 10^6^ CFU/mL) at a dose of 500 μL/100 g body mass. Transcription was normalized against unchallenged samples. **(A)** Transcripts of *Lmcxcl8* detected in the intestine and kidney (Kruskal–Wallis test, *p* < 0.01; Dunn multiple comparisons), and SB and gill (one-way ANOVA, *p* < 0.0001; Tukey multiple comparisons). **(B)** Transcripts of *Lmcxcl17* detected in the intestine, kidney, SB, and gill (one-way ANOVA, *p* < 0.0001; Tukey multiple-comparisons test). β*-Actin* transcripts were used as an internal control for real-time PCR. Bars show mean ± SEM of data from four lampreys pooled from three independent experiments. Lowercase letters above each bar denote statistical results from multiple comparisons: the bars sharing the same letter represent means that are not different from each other (*p* > 0.05), whereas the bars labeled with different letters indicate means that are different (*p* < 0.05).

Substantial increases of *Lmcxcl8* were detected in the intestine, kidney, SB, and gill in adult lampreys at 6 h after intraperitoneal injection of the bacteria. The *Lmcxcl8* level peaked at 12 h in the gill and SB and at 24 h in the kidney and intestine (Figure 5A). Likewise, the *Lmcxcl17* level peaked at 24 h in the kidney, SB, gill, and intestine (Figure 5B). Our results showed that the expression of *Lmcxcl8* gene was inducible by bacterial challenge in the larvae and adults.

### Chemotaxis Induced by LmCXCL8

**Figure 6:**
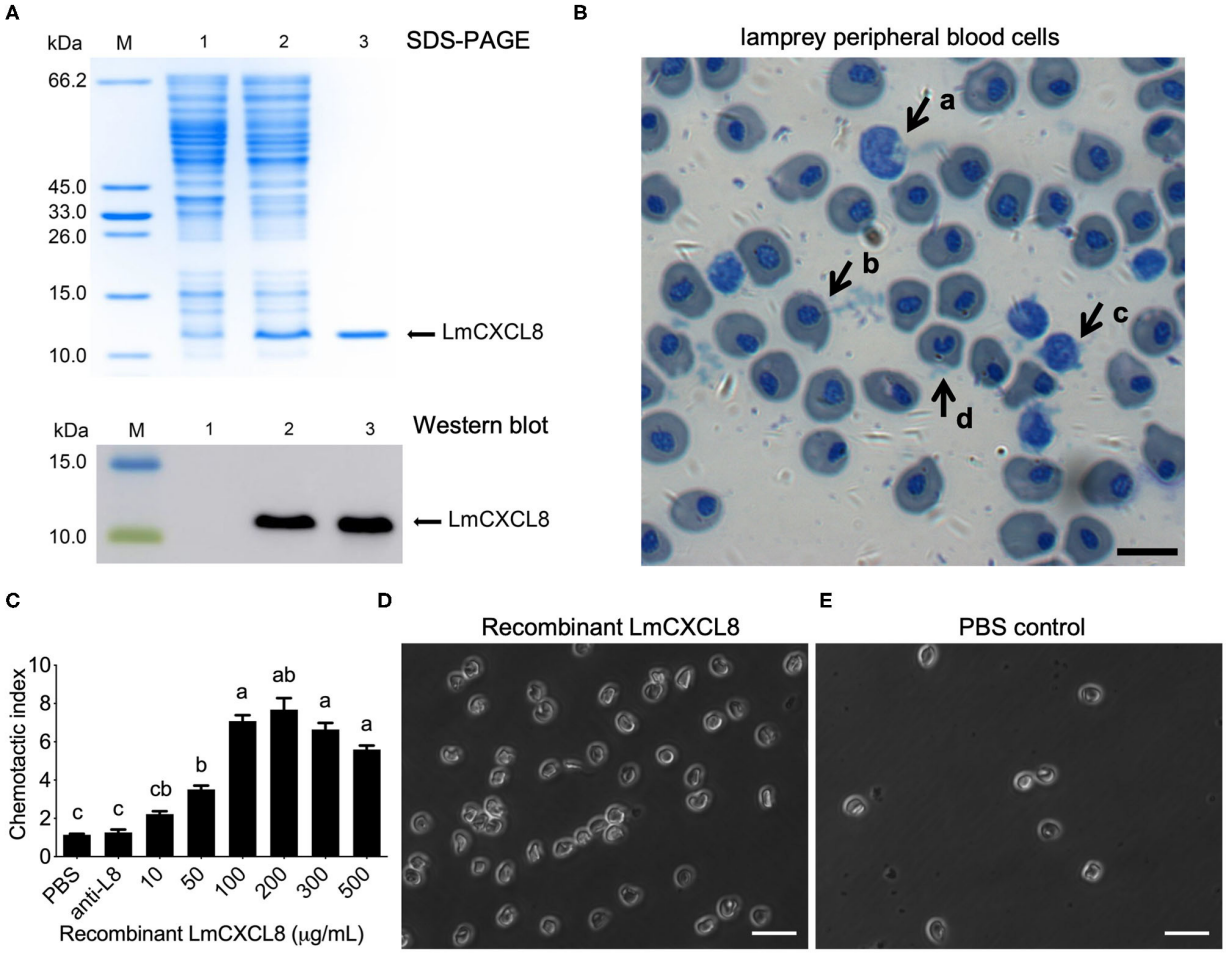
Recombinant LmCXCL8 induces chemotaxis in lamprey leukocytes. **(A)** Sodium dodecyl sulfate–PAGE and Western blot analysis of recombinant LmCXCL8 protein. M: protein molecular weight marker. Lane 1: induced pColdI/BL21 (as a control); Lane 2: induced pColdI-CXCL8/BL21; Lane 3: purified recombinant LmCXCL8 protein. **(B)** Microscopic structures of the lamprey peripheral blood cells (×400). Wright-stained smear showing the lamprey peripheral blood cells. The scale bar is 10 μm. (a) Monocytes, (b) erythrocyte, (c) lymphocyte, (d) neutrophil. **(C)** *In vitro* chemotaxis of lamprey leukocytes to recombinant LmCXCL8. Brown–Forsythe and Welch ANOVA test, *p* < 0.0001; lowercase letters above each bar denote statistical results from Dunnett T3 multiple-comparisons test: the bars sharing the same letter represent means that are not different from each other (*p* > 0.05), whereas the bars labeled with different letters indicate means that are different (*p* < 0.05). One hundred microliters of PBS was used as a negative control. Anti-L8, anti-CXCL8 antibody-blocking treatment for 4 h. Data indicate the mean ± SEM from three lampreys pooled from three independent experiments. **(D)** *In vitro* chemotaxis of lamprey leukocytes that migrated into lower chamber contains recombinant CXCL8 protein (200 μg/mL). The scale bar is 20 μm. **(E)** *In vitro* chemotaxis of lamprey leukocytes that migrate into lower chamber contains phosphate-buffered saline (PBS). The scale bar is 20 μm.

To assess the chemotactic function of LmCXCL8, we first produced a recombinant LmCXCL8. Sodium dodecyl sulfate–PAGE and Western blot analysis showed that the purified recombinant protein occurred as a single band with a molecular mass of roughly 11.2 kDa (Figure 6A). Matrix-assisted laser desorption/ionization–TOF/TOF analyses showed that the recombinant LmCXCL8 was identical to a peptide sequence predicted from *Lmcxcl8* (Supplementary Figures 1A,B).

Monocytes, erythrocytes, lymphocytes, and neutrophils were found in the lamprey PBLs (Figure 6B). Peripheral blood leukocytes were attracted to the recombinant LmCXCL8 in a concentration-dependent manner (Figure 6C). Within the range between 10 and 500 μg/mL, recombinant LmCXCL8 showed peak chemotactic activity at 200 μg/mL (Figures 6C,D). The PBS control and the mixture of the blocking antibody with the recombinant LmCXCL8 did not induce chemotaxis in lamprey PBLs (Figures 6C,E).

## Discussion

Our results are consistent with the hypothesis that chemokine induces homologous chemotaxis in basal vertebrates. First, we identified LmCXCL8 based on its typical arrangement of four conserved cysteine residues and CXC cysteine-motif of CXC chemokine, and its homology to previously reported CXCL8 of other vertebrate species in phylogenetic analyzes. Second, its expression and distribution patterns are consistent with the predicted function of a chemokine. In addition, the recombinant LmCXCL8 protein induces chemotactic response in Lamprey PBLs, tracing the chemokine-induced chemotaxis to a basal vertebrate. These results, while providing insights into the evolution of interactions between PBL and chemokine, are also consistent with the notion that innate immunity evolved much earlier than the acquired immunity.

The expression pattern of *Lmcxcl8* showed unique characteristics. In several healthy lampreys, *Lmcxcl8* was detected at high levels in spleen, kidney, liver, and gill, which is consistent with the constitutive expression pattern of *cxcl8* in jawed fish species (27, 37–39). The strong expression of *Lmcxcl8* in SB support that SB is a crucial immune tissue of the immune defense system in adult lampreys (40). The strong expression of *Lmcxcl8* in SB also supports the potential hematopoietic function of SB. CXCL8 was reported to promote hematopoietic stem cell production via promoting the impact of active vitamin D_3_ (41), and indeed, hematopoietic cells exist in SB of postmetamorphic lamprey (42). It would be interesting to determine if high expression levels of *Lmcxcl8* are due to massive numbers of resident leukocytes in SB tissues.

The inducible expression of *Lmcxcl8* suggests that it may play a significant role in inflammation. In teleost, CXCL8 is known to play a critical role in inflammatory responses (43). The expression of *cxcl8* was up-regulated significantly upon stimulation by bacteria in a number of fishes (27). Similarly, CXCL17 is also an important proinflammatory chemokine in lamprey (11, 12). Our data show that, *P. aeruginosa*, isolated from diseased Northeast Chinese lamprey (unpublished data), induced rapid and strong up-regulation of expression of *Lmcxcl8* and *Lmcxcl17* in both juveniles and adults. Our results provide strong evidence that LmCXCL8 takes part in innate immune response against bacterial infection in lamprey. After the entry of pathogen into an individual, CXCL8 is regulated primarily at the level of gene transcription. Transcription factors such as activator protein 1, nuclear factor–IL-6 (44), and nuclear factor κB (NF-κB) (45), as well as cytokine TNF-α (46), induce *cxcl8* transcription. The secreted CXCL8 recruit leukocytes (mainly neutrophils) that express its receptors CXCR1 and CXCR2 (CXCR1/2). When binding with CXCL8, CXCR1/2 activate the G protein cascades and activate a series of transcription factors, such as AP-1 (47), androgen receptor, NF-κB, signal transducer, and activator of transcription 3, as well as β-catenin (48) and intercellular adhesion molecule 1 (49). These events upregulate gene expressions associated with cell immigration, survival, angiogenesis, invasion, proliferation, and metabolism. However, only NF-κB of the aforementioned upstream and downstream gene of CXCL8 was identified in lamprey (50). Also, it is essential to identify the LmCXCL8 receptor and to define its role in the chemotactic effect mechanism of LmCXCL8 and understand how it interacts with the LmCXCL8 GGR motif in the future.

The increased level of LmCXCL8 upon bacteria challenge has been proposed to provide a useful mechanism for this chemokine to recruit PBLs to sites of inflammation. In mammals, it is well-established that CXCL8 plays an important role in oriented migration of leukocytes, promoting a positive chemotaxis toward sites of inflammation (51). Likewise, chemotactic activities of recombinant CXCL8 have been reported in common carp (28), zebrafish (52), rainbow trout (26), Japanese flounder (29), and large yellow croaker (30). In our study, the recombinant LmCXCL8 protein exhibited obvious chemotactic activities for lamprey PBLs at an optimum concentration of 200 μg/mL. Notably, a decrease in chemotaxis was observed at higher concentration from 300 to 500 μg/mL, probably due to receptor desensitization, as has been commonly observed for chemokines (53–55). The optimum concentration of recombinant LmCXCL8 protein was not substantially different from those of teleost fishes, such as Japanese flounder (100 ng/mL/mL) (29), rainbow trout (150 ng/mL) (26), and large yellow croaker (100 μg/mL) (30). The chemotactic index and action time in the lamprey are similar to those reported for fish CXCL8. Additionally, PBLs lost chemotactic response when LmCXCL8 was blocked by a specific antibody. Hence, we confirmed the lamprey PBLs were attracted to the recombinant LmCXCL8 in a concentration-dependent manner at the help of specific LmCXCL8 polyantibody. These data demonstrated that LmCXCL8 is sufficient for chemotaxis of PBLs.

Our data also suggested that the GGR motif is sufficient for LmCXCL8 to exert its chemotaxis function, as has been found in most known fish CXCL8 lack of complete ELR motif (13). The ELR motif of CXCL8 plays an important role in attracting neutrophils and angiogenesis in mammals (56). LmCXCL8, like most fish CXCL8, does not have the ELR motif; instead, it has a GGR motif. Previous phylogenetic studies suggest that the ELR-like motif of ancestor fish had evolved from the GGR of Lamprey (31, 32). Our data further suggest the GGR motif is a functional ancestor to the ELR-like motif because recombinant LmCXCL8 readily induces chemotaxis in lamprey PBLs.

Our cloning and phylogenic analyses demonstrate that LmCXCL8 is homologous to CXCL8 of other vertebrate species. The deduced LmCXCL8 possesses four conserved cysteines, which are essential for the tertiary structure and function of CXC chemokine (57). Its first two cysteine residues near the N-terminal are separated by a single glutamine residue, placing it into the CXC chemokines subfamily (13). Using SAMART program, we predicted that the first 23 aa at N-terminal from a signal peptide. An SCY domain, which is characteristic of secretory chemokines (58), was also found in LmCXCL8. Also, the lamprey CXCL8 clade is positioned between spotted gar CXCL8 and coelacanth CXCL8 as outbranches of teleost CXCL8-L1, as an outbranch of teleost CXCL8-L1 consists in the phylogenetic tree in previous report (31). Besides, in our phylogenetic analyses, teleost CXCL8-L2 grouped with the clades of bird and mammal, separated from teleost CXCL8-L1 and CXCL8-L3 groups, which supported the previous studies (1, 30, 59). In addition, we found that most teleost species possessed two CXCL8 and were distributed in the CXCL8-L1 and CXCL8-L3, whereas only cyprinid fish species (common carp, grass carp, and zebrafish) had CXCL8 that were distributed in the CXCL8-L2. We speculate that the teleost CXCL8-L1 and CXCL8-L3 groups represent the teleost CXCL8 derived from the teleost-specific genome duplication (3R). Cyprinid fish CXCL8-L2 clade may represent a rapid rate of evolution, which leads to close clustering to the CXCL8 in birds and mammals.

In conclusion, LmCXCL8 is homologous to those of many other species, in both structures and functions. LmCXCL8 likely plays an important role in lamprey innate immune response.

## Data Availability Statement

The datasets generated for this study can be found in UniProt-the full-length cDNA of Lmcxcl8 gene from Northeast Chinese lamprey (accession number: KY379068).

## Ethics Statement

The animal study was reviewed and approved by Shanghai Ocean University Experimentation Ethics Review Committee.

## Author Contributions

QZ and WL conceived the project. QZ designed the experiments. XZ performed the all of the experiments and analyzed the data. ZZ and SD performed the experiments. ZZ cloned the *Lmcxcl8* gene, performed the phylogenetic analysis, and statistical analysis. JR provided the sequence of *Lmcxcl8* gene. WM provided *P. aeruginosa* strain PA11. LJ provided Northeast Chinese lamprey. YT provided the methods for expression and purification of protein. XZ and ZZ wrote the paper. QZ, WL, XZ, and ZZ reviewed and edited the manuscript. YZ and JP provided useful advice. All authors contributed to the article and approved the submitted version.

## Conflict of Interest

The authors declare that the research was conducted in the absence of any commercial or financial relationships that could be construed as a potential conflict of interest.

## Abbreviations

LmCXC8: CXCL8 in Northeast Chinese lamprey
LmCXC17: CXCL17 in Northeast Chinese lamprey
SB: supraneural body.

## References

[1] Chen J Xu Q Wang T Collet B Corripio-Miyar Y Bird S. Phylogenetic analysis of vertebrate CXC chemokines reveals novel lineage specific groups in teleost fish. Dev Comp Immunol. (2013) 41:137–52. 10.1016/j.dci.2013.05.006 23701879

[2] Kuroda N Uinukool TS Sato A Samonte IE Figueroa F Mayer WE. Identification of chemokines and a chemokine receptor in cichlid fish, shark, and lamprey. Immunogenetics. (2003) 54:884–95. 10.1007/s00251-002-0531-z 12671740

[3] Alejo A Tafalla C. Chemokines in teleost fish species. Dev Comp Immunol. (2011) 35:1215–22. 10.1016/j.dci.2011.03.011 21414348

[4] Van Haastert PJ Devreotes PN. Chemotaxis: signalling the way forward. Nat Rev Mol Cell Biol. (2004) 5:626–34. 10.1038/nrm1435 15366706

[5] Zlotnik A Yoshie O. The chemokine superfamily revisited. Immunity. (2012) 36:705–16. 10.1016/j.immuni.2012.05.008 22633458 PMC3396424

[6] Esche C Stellato C Beck LA. Chemokines: key players in innate and adaptive immunity. J Invest Dermatol. (2005) 125:615–28. 10.1111/j.0022-202X.2005.23841.x 16185259

[7] Kunkel SL Strieter RM Lindley IJD Westwick J. Chemokines: new ligands, receptors and activities. Immunol Today. (1995) 16:559–61. 10.1016/0167-5699(95)80076-X 8579746

[8] Himmel ME Crome SQ Ivison S Piccirillo C Steiner TS Levings MK. Human CD4+ FOXP3+ regulatory T cells produce CXCL8 and recruit neutrophils. Eur J Immunol. (2011) 41:306–12. 10.1002/eji.201040459 21268001

[9] Sokol CL Luster AD. The chemokine system in innate immunity. Cold Spring Harbor Pers Biol. (2015) 7:a016303. 10.1101/cshperspect.a016303 25635046 PMC4448619

[10] Najakshin AM Mechetina LV Alabyev BY Taranin AV. Identification of an IL-8 homolog in lamprey (*Lampetra fluviatilis*): early evolutionary divergence of chemokines. Eur J Immunol. (1999) 29:375–82. 10.1002/(SICI)1521-4141(199902)29:02<375::AID-IMMU375>3.0.CO;2-6 10064052

[11] Tsutsui S Nakamura O Watanabe T. Lamprey (*Lethenteron japonicum*) IL-17 upregulated by LPS-stimulation in the skin cells. Immunogenetics. (2007) 59:873–82. 10.1007/s00251-007-0254-2 17924104

[12] Han Q Das S Hirano M Holland SJ McCurley N Guo P. Characterization of lamprey IL-17 family members and their receptors. J Immunol. (2015) 195:5440–51. 10.4049/jimmunol.1500892 26491201 PMC4655163

[13] Laing KJ Secombes CJ. Chemokines. Dev Comp Immunol. (2004) 28:443–60. 10.1016/j.dci.2003.09.006 15062643

[14] Bickel M. The role of interleukin-8 in inflammation and mechanisms of regulation. J Periodontol. (1993) 64:456–60. 8315568

[15] Srivastava V Dey I Leung P Chadee K. Prostaglandin E2 modulates IL-8 expression through formation of a multiprotein enhanceosome in human colonic epithelial cells. Eur J Immunol. (2012) 42:912–23. 10.1002/eji.201141965 22531917

[16] Eckmann L Kagnoff MF Fierer J. Epithelial cells secrete the chemokine interleukin-8 in response to bacterial entry. Infect Immunity. (1993) 61:4569–74. 10.1128/IAI.61.11.4569-4574.1993 8406853 PMC281206

[17] Larsson BM Larsson K Malmberg P Palmberg L. Gram positive bacteria induce IL-6 and IL-8 production in human alveolar macrophages and epithelial cells. (1999) 23:217–30. 10392756 10.1023/a:1020269802315

[18] Hirao Y Kanda T Aso Y Mitsuhashi M Kobayashi I Interleukin-8—an early marker for bacterial infection. Lab Med. (2000) 31:39–44. 10.1309/GJ98-JAH8-VQ57-D6N0

[19] Horne NS Shaw H Huang Y Frieri M Modulation of IL8 mRNA expression and production in respiratory syncytial virus (RSV) stimulated human alveolar epithelial cells by budesonide and montelukast. J Allergy Clin Immunol. (2004) 113:S192 10.1016/j.jaci.2004.01.132

[20] Lane BR Lore K Bock PJ Andersson J Coffey MJ Strieter RM. Interleukin-8 stimulates human immunodeficiency virus type 1 replication and is a potential new target for antiretroviral therapy. J Virol. (2001) 75:8195–202. 10.1128/JVI.75.17.8195-8202.2001 11483765 PMC115064

[21] Qazi BS Kai T Qazi A. Recent advances in underlying pathologies provide insight into interleukin-8 expression-mediated inflammation and angiogenesis. Int J Inflamm. (2011) 2011:908468. 10.4061/2011/908468 22235381 PMC3253461

[22] Mukaida N. Interleukin-8: an expanding universe beyond neutrophil chemotaxis and activation. Int J Hematol. (2000) 72:391−8. 11197203

[23] Wu YF Shien JH Yin HH Chiow SH Long HL. Structural and functional homology among chicken, duck, goose, turkey and pigeon interleukin-8 proteins. Vet Immunol Immunopathol. (2008) 125:205–15. 10.1016/j.vetimm.2008.03.001 18757102

[24] Cui X Han Y Pan Y Xu X Ren W Zhang S. Molecular cloning, expression and functional analysis of interleukin-8 (IL-8) in South African clawed frog (*Xenopus laevis*). Dev Comp Immunol. (2011) 35:1159–65. 10.1016/j.dci.2011.04.005 21530580

[25] DeVries ME Kelvin AA Xu L Ran L Robinson J Kelvin DJ. Defining the origins and evolution of the chemokine/chemokine receptor system. J Immunol. (2005) 176:401–15. 10.4049/jimmunol.176.1.401 16365434

[26] Montero J Coll J Sevilla N Cuesta A Bols NC Tafalla C. Interleukin 8 and CK-6 chemokines specifically attract rainbow trout (*Oncorhynchus mykiss*) RTS11 monocyte-macrophage cells and have variable effects on their immune functions. Dev Comp Immunol. (2008) 32:1374–84. 10.1016/j.dci.2008.05.004 18572244

[27] Sun JS Zhao L Sun L. Interleukin-8 of *Cynoglossus semilaevis* is a chemoattractant with immunoregulatory property. Fish Shellfish Immunol. (2011) 30:1362–7. 10.1016/j.fsi.2011.03.023 21496489

[28] van der Aa LM Chadzinska M Golbach LA Ribeiro CM Lidy Verburg-van Kemenade BM. Pro-inflammatory functions of carp CXCL8-like and CXCb chemokines. Dev Comp Immunol. (2012) 36:741–50. 10.1016/j.dci.2011.11.011 22182503

[29] Kurata O Wada S Matsuyama T Sakai T Takano T. N-Terminal region is responsible for chemotaxis-inducing activity of flounder IL-8. Fish Shellfish Immunol. (2014) 38:361–6. 10.1016/j.fsi.2014.04.006 24751922

[30] Mu Y Wang K Ao J Chen X. Molecular characterization and biological effects of a CXCL8 homologue in large yellow croaker (*Larimichthys crocea*). Fish Shellfish Immunol. (2015) 44:462–70. 10.1016/j.fsi.2015.03.026 25827624

[31] Gangele K Jamsandekar M Mishra A Poluri KM. Unraveling the evolutionary origin of ELR motif using fish CXC chemokine CXCL8. Fish Shellfish Immunol. (2019) 93:17–27. 10.1016/j.fsi.2019.07.034 31310848

[32] Zhonghua C Chunpin G Yong Z Kezhi X Yaou Z. Cloning and bioactivity analysis of a CXC ligand in black seabream *Acanthopagrus schlegeli*: the evolutionary clues of ELR+CXC chemokines. BMC Immunol. (2008) 9:66. 10.1186/1471-2172-9-66 18990254 PMC2585555

[33] Shimeld SM Donoghue PC. Evolutionary crossroads in developmental biology: cyclostomes (lamprey and hagfish). Development. (2012) 139:2091–9. 10.1242/dev.074716 22619386

[34] Tamura K Stecher G Peterson D Filipski A Kumar S. MEGA6: molecular evolutionary genetics analysis version 6.0. Mol Biol Evol. (2013) 30:2725–9. 10.1093/molbev/mst197 24132122 PMC3840312

[35] Yue P Qingwei L Microscopic structures of Japanese lamprey peripheral blood cells and culture of lymphocyte-like cells *in vitro*. Marine Sciences. (2012) 36:23–9. 10.1073/pnas.212527499

[36] Robert X Gouet P. Deciphering key features in protein structures with the new ENDscript server. Nucleic Acids Res. (2014) 42:W320–4. 10.1093/nar/gku316 24753421 PMC4086106

[37] Corripio-Miyar Y Bird S Tsamopoulos K Secombes CJ. Cloning and expression analysis of two pro-inflammatory cytokines, IL-1 beta and IL-8, in haddock (*Melanogrammus aeglefinus*). Mol Immunol. (2007) 44:1361–73. 10.1016/j.molimm.2006.05.010 16831460

[38] Hu YH Chen L Sun L. CXCL8 of scophthalmus maximus: expression, biological activity and immunoregulatory effect. Dev Comp Immunol. (2011) 35:1032–9. 10.1016/j.dci.2011.04.002 21530579

[39] Laing KJ Zou JJ Wang T Bols N Hirono I Aoki T. Identification and analysis of an interleukin 8-like molecule in rainbow trout *Oncorhynchus mykiss*. Dev Comp Immunol. (2002) 26:433–44. 10.1016/S0145-305X(01)00092-1 11906723

[40] Pang Y Li C Wang S Ba W Yu T Pei G A novel protein derived from lamprey supraneural body tissue with efficient cytocidal actions against tumor cells. Cell Commun Signal. (2017) 15:42 10.1186/s12964-017-0202-1 29037260 PMC5644163

[41] Cortes M Chen MJ Stachura DL Liu SY Kwan W Wright F. Developmental vitamin D availability impacts hematopoietic stem cell production. Cell Rep. (2016) 17:458–68. 10.1016/j.celrep.2016.09.012 27705794 PMC5338633

[42] Amemiya CT Saha NR Zapata A. Evolution and development of immunological structures in the lamprey. Curr Opin Immunol. (2007) 19:535–41. 10.1016/j.coi.2007.08.003 17875388 PMC2093943

[43] Magnadottir B. Innate immunity of fish (overview). Fish Shellfish Immunol. (2006) 20:137–51. 10.1016/j.fsi.2004.09.006 15950491

[44] Lin CH Cheng HW Ma HP Wu CH Hong CY Chen BC. Thrombin induces NF-κB activation and IL-8/CXCL8 expression in lung epithelial cells by a Rac1-dependent PI3K/Akt pathway. J Biol Chem. (2011) 286:10483–94. 10.1074/jbc.M110.112433 21266580 PMC3060502

[45] Khalaf H Jass J Olsson PE. The role of calcium NF-κB NFAT in the regulation of CXCL8 IL-6 expression in Jurkat T-cells. Int J Biochem Mol Biol. (2013) 4:150−6. 24049670 PMC3776147

[46] Namba S Nakano R Kitanaka T Kitanaka N Nakayama T Sugiya H. ERK2 and JNK1 contribute to TNF-α-induced IL-8 expression in synovial fibroblasts. PLoS ONE. (2017) 12:e0182923. 10.1371/journal.pone.0182923 28806729 PMC5555573

[47] Walana W Wang JJ Yabasin IB Ntim M Kampo S Al-Azab M. IL-8 analogue CXCL8 (3-72) K11R/G31P, modulates LPS-induced inflammation via AKT1-NF-kβ and ERK1/2-AP-1 pathways in THP-1 monocytes. Hum Immunol. (2018) 79:809–16. 10.1016/j.humimm.2018.08.007 30125599

[48] Waugh DJ Wilson C. The interleukin-8 pathway in cancer. Clin Cancer Res. (2008) 14:6735–41. 10.1158/1078-0432.CCR-07-4843 18980965

[49] Citro A Cantarelli E Piemonti L. The CXCR1/2 pathway: involvement in diabetes pathophysiology and potential target for T1D interventions. Curr Diab Rep. (2015) 15:68. 10.1007/s11892-015-0638-x 26275440

[50] Su P Liu X Pang Y Liu C Li R Zhang Q. The archaic roles of the lamprey NF-κB (lj-NF-κB) in innate immune responses. Mol Immunol. (2017) 92:21–7. 10.1016/j.molimm.2017.10.002 29031044

[51] Lin F Nguyen CM Wang SJ Saadi W Gross SP Jeon NL. Effective neutrophil chemotaxis is strongly influenced by mean IL-8 concentration. Biochem Biophys Res Commun. (2004) 319:576–81. 10.1016/j.bbrc.2004.05.029 15178445

[52] de Oliveira S Reyes-Aldasoro CC Candel S Renshaw SA Mulero V Calado A. Cxcl8 (IL-8) mediates neutrophil recruitment and behavior in the zebrafish inflammatory response. J Immunol. (2013) 190:4349–59. 10.4049/jimmunol.1203266 23509368 PMC3736093

[53] Ferguson SS. Evolving concepts in G protein-coupled receptor endocytosis: the role in receptor desensitization and signaling. Pharmacol Rev. (2001) 53:1–24. 11171937

[54] Johnston JA Ferris DK Wang JM Longo DL Oppenheim JJ Kelvin DJ. Staurosporine restores signaling and inhibits interleukin-8-induced chemotactic desensitization. Eur J Immunol. (1994) 24:2556–62. 10.1002/eji.1830241044 7925583

[55] Barlic J Khandaker MH Mahon E Andrews J Devries ME Mitchell GB. β-arrestins regulate interleukin-8-induced CXCR1 internalization. J Biol Chem. (1999) 274:16287. 10.1074/jbc.274.23.16287 10347185

[56] Wuyts A Proost P Lenaerts JP Ben-Baruch A Van DJ Wang JM. Differential usage of the CXC chemokine receptors 1 and 2 by interleukin-8, granulocyte chemotactic protein-2 and epithelial-cell-derived neutrophil attractant-78. (1998) 255:67–73. 10.1046/j.1432-1327.1998.2550067.x 9692902

[57] Zlotnik A Yoshie O. Chemokines: a new classification system and their role in immunity. Immunity. (2000) 12:121–7. 10.1016/S1074-7613(00)80165-X 10714678

[58] And EJF Lolis E Structure, function, and inhibition of chemokines. Annu Rev Pharmacol Toxicol. (2002) 42:469–99. 10.1146/annurev.pharmtox.42.091901.115838 11807180

[59] van der Aa LM Chadzinska M Tijhaar E Boudinot P Verburg-van Kemenade BM. CXCL8 chemokines in teleost fish: two lineages with distinct expression profiles during early phases of inflammation. PLoS ONE. (2010) 5:e12384. 10.1371/journal.pone.0012384 20865040 PMC2928728

