# Supplementary material for: Molecular Characterization and Chemotactic Function of CXCL8 in Northeast Chinese Lamprey (Lethenteron morii)
Source: Front Immunol. 2020 Aug 18;11:1738. doi: 10.3389/fimmu.2020.01738 (PMC7461807; doi:10.3389/fimmu.2020.01738)
Supplement: Supplementary file 1 [file Data_Sheet_1.PDF]

SUPPLEMENTARY MATERIAL

Molecular characterization and chemotactic function of CXCL8 in Northeast Chinese lamprey (*Lethenteron morii*)

Running title: Lamprey CXCL8 characteristics and function

Xinyun Zhu[a,b,c,1], Zhe Zhang[a,b,c,1], Jianfeng Ren[a,b,c,], Liang Jia[a,b,c], Shaoqing Ding[a,b,c], Jiafei Pu[a,b,c], Wenyuan Ma[a,b,c], Yan Tao[d], Yao Zu[a,b,c], Weiming Li[e, *], Qinghua Zhang[a,b,c,*]

[a] Key Laboratory of Exploration and Utilization of Aquatic Genetic Resources, Ministry of Education, Shanghai Ocean University, Shanghai 201306, China

[b] International Research Center for Marine Biosciences, Ministry of Science and Technology, Shanghai Ocean University, Shanghai 201306, China

[c] Key Laboratory of Freshwater Aquatic Genetic Resources, Ministry of Agriculture, Shanghai Ocean University, Shanghai 201306, China

[d] College of Food Science and Technology, Shanghai Ocean University, Shanghai 201306, China

[e] Department of Fisheries and Wildlife, Michigan State University, East Lansing, MI 48824, USA

*Correspondence:

Dr. Qinghua Zhang

Email: qhzhang@shou.edu.cn

Dr. Weiming Li

Email: liweim@msu.edu

[1]These authors contributed equally to this work.

SUPPLEMENTARY MATERIAL

A

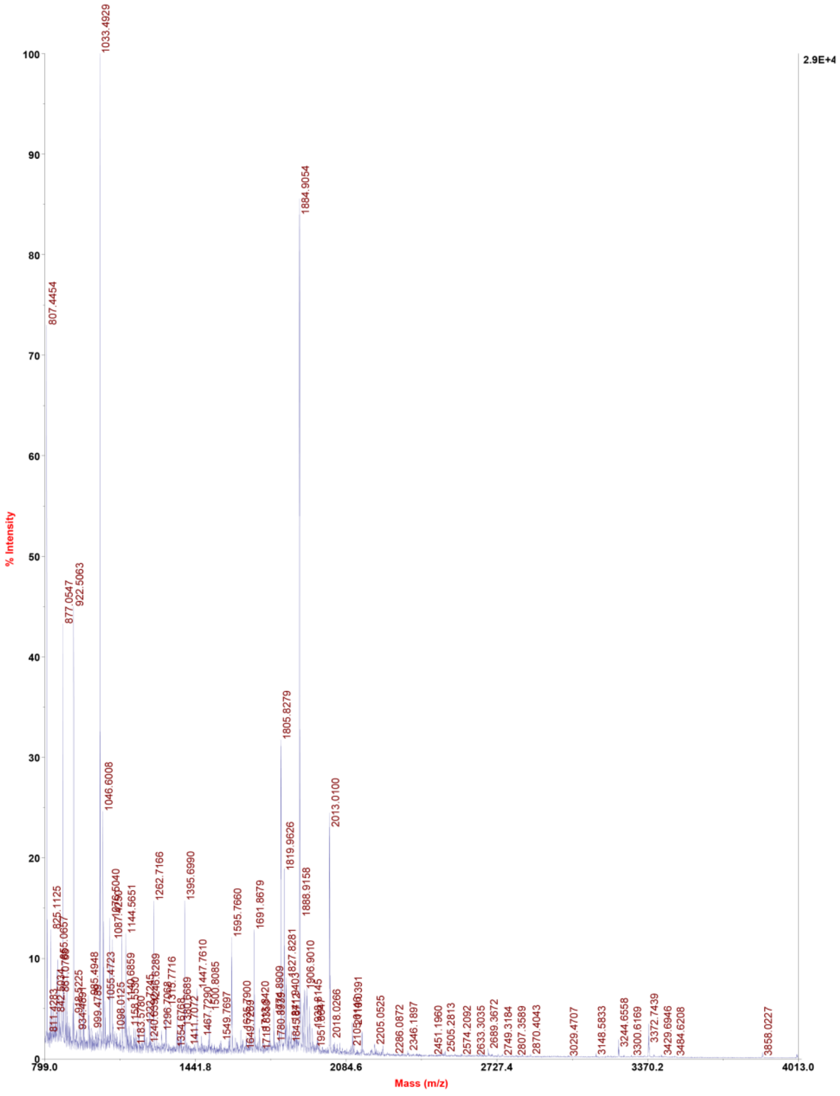

B

Calc. Mass	Obsrv. Mass	± da	± ppm	Start Seq.	End Seq.	Sequence	Ion Score	C. I. %	Modification	Rank	Result Type
896.4294	896.4327	0.0033	4	1	8	MSIFEGGR					Mascot
1046.5914	1046.6013	0.0099	9	38	46	NVEIIVTMK					Mascot
1046.5914	1046.6013	0.0099	9	38	46	NVEIIVTMK	12	99.695			Mascot
1144.5602	1144.5653	0.0051	4	9	17	CQCIHVISK			Carbamidomethyl (C)[1,3]		Mascot
1691.8785	1691.8685	-0.01	-6	64	79	VMSHILDGAQTPKPTP					Mascot
1691.8785	1691.8685	-0.01	-6	64	79	VMSHILDGAQTPKPTP	79	100			Mascot
1805.8309	1805.828	-0.0029	-2	23	37	HFQTMEVIPQSSNCK			Carbamidomethyl (C)[14]		Mascot
1805.8309	1805.828	-0.0029	-2	23	37	HFQTMEVIPQSSNCK	109	100	Carbamidomethyl (C)[14]		Mascot
1819.9735	1819.9626	-0.0109	-6	63	79	KVMSHILDGAQTPKPTP					Mascot
1819.9735	1819.9626	-0.0109	-6	63	79	KVMSHILDGAQTPKPTP	41	100			Mascot
1827.8806	1827.8281	-0.0525	-29	47	62	STNNQICLNPDAPWVR					Mascot
1835.9685	1835.9396	-0.0289	-16	63	79	KVMSHILDGAQTPKPTP			Oxidation (M)[3]		Mascot
1884.9021	1884.9056	0.0035	2	47	62	STNNQICLNPDAPWVR			Carbamidomethyl (C)[7]		Mascot
1884.9021	1884.9056	0.0035	2	47	62	STNNQICLNPDAPWVR	116	100	Carbamidomethyl (C)[7]		Mascot
2012.9971	2013.0084	0.0113	6	47	63	STNNQICLNPDAPWVRK			Carbamidomethyl (C)[7]		Mascot
2012.9971	2013.0084	0.0113	6	47	63	STNNQICLNPDAPWVRK	42	100	Carbamidomethyl (C)[7]		Mascot

SUPPLEMENTARY FIGURE 1 | MALDI-TOF/TOF spectra of LmCXCL8 and the results of database retrieval. **(A)** MALDI-TOF/TOF spectra of PmCXCL8. **(B)** The results of database retrieval.
